# Associations between Consumption of Dairy Foods and Anthropometric Indicators of Health in Adolescents

**DOI:** 10.3390/nu8070427

**Published:** 2016-07-13

**Authors:** Manijeh Nezami, Gina Segovia-Siapco, W. Lawrence Beeson, Joan Sabaté

**Affiliations:** Center for Nutrition, Healthy Lifestyles, and Disease Prevention, Loma Linda University, Loma Linda, CA 92350, USA; mnezami@llu.edu (M.N.); lbeeson@llu.edu (W.L.B.); jsabate@llu.edu (J.S.)

**Keywords:** dairy, adolescents, anthropometric, milk, BMI z-score, waist to height ratio

## Abstract

Childhood obesity is associated with a greater chance of a lifetime of obesity. Evidence suggests dairy at recommended levels could be beneficial in maintaining normal weight and body composition. We assessed whether dairy consumption is associated with anthropometric indicators of health (z-scores for weight-for-age (WAZ); height-for-age (HAZ) and body mass index (BMIZ); waist-to-height ratio (WHtR); fat-free mass (FFM); and fat mass (FM)) in adolescents. In a cross-sectional study, 536 males and females ages 12–18 completed a 151-item semi-quantitative web-based food frequency questionnaire that included 34 dairy-containing foods. Dairy foods were categorized into milk, cheese, sweetened dairy, and total dairy. Anthropometrics were measured during school visits. Total dairy intake was associated with WAZ (β = 0.25 (95% CI: 0.01, 0.49), *p* = 0.045) and HAZ (β = 0.28 (95% CI: 0.04, 0.52), *p* = 0.021). In boys, total dairy was associated with WHtR (β = 0.02 (95% CI: 0.00, 0.04), *p* = 0.039), FFM (β = 4.83 (95% CI: 1.79, 7.87), *p* = 0.002), and FM (β = 3.89 (95% CI: 0.58, 7.21), *p* = 0.021), and cheese was associated with FFM (β = 4.22 (95% CI: 0.98, 7.47), *p* = 0.011). Dairy consumption seems to influence growth in both genders, and body composition and central obesity in boys. Prospective studies are needed to identify how types of dairy relate to growth, body composition, and central obesity of adolescents.

## 1. Introduction

Child and adolescent obesity continues to be a significant public health challenge [1,2]. Diet is the most important lifestyle factor associated with overweight and obesity [2,3]. The Dietary Guidelines for Americans and My Plate recommendations were developed to help consumers choose from each food group wisely. However, we continue to observe a large disparity between the recommendations and what is actually consumed, particularly among the adolescent population [3,4,5,6].

Milk and dairy products comprise one food group long advertised as an excellent source of nutrition [7]. Dairy products provide a rich source of many nutrients including protein, calcium, magnesium, and vitamins A and B [8,9]. Calcium is one of the key nutrients believed to be responsible for the positive findings between dairy and prevention of weight gain [10,11,12].

During the last decade, over a dozen studies found that higher intakes of milk or dairy calcium may help in preventing weight gain and/or changes in body composition [13,14,15,16,17,18,19,20,21,22]. However, other studies show no relationship between dairy consumption and body weight or fat percentages [23,24,25,26,27]. This has raised a considerable controversy about whether or not the amount or level of dairy intake is associated with loss or gain of body weight or fat.

Because of this disparity between studies, milk and dairy are widely studied and continue to be one of the most controversial food groups, especially in relation to its potential connection with overweight and obesity in children and adolescents [6,28,29]. Lactose intolerance, potential allergic reactions, and/or concerns over growth hormones and antibiotic use in cows are other reasons cited for not drinking milk [8,30,31].

The Adventist population has been the focus of multiple epidemiologic studies due to their diverse dietary habits [32] and adherence to a healthy lifestyle which includes avoiding smoking and alcohol [33,34]. The Adventist population’s milk and dairy food habits are especially varied due to a diverse dietary practice ranging from omnivorous to vegetarian [35,36]. This makes the Adventist population very appealing to study with the intention of better understanding diet patterns and creating better dietary guidelines for adolescents. Furthermore, since no studies were done on an adolescent population known to subscribe to the Adventist lifestyle and diet, it is worth investigating whether dairy intake in this group is related to their growth and/or risks for overweight/obesity.

The purpose of our study was to determine the interrelationships between milk and dairy product consumption and growth parameters (z-scores for weight-for-age (WAZ), height-for-age (HAZ), and BMI (BMIZ)); indicators of overweight/obesity (BMIZ, waist-to- height ratio (WHtR), and waist circumference); and body composition indicators (fat-free mass and fat mass) in an Adventist adolescent population.

## 2. Materials and Methods

### 2.1. Study Design and Participants

The Teen Food and Development Study (TFADS) is a cross-sectional study designed to ascertain associations among types of foods consumed, physical growth, and pubertal development [37]. Study participants were recruited from Adventist and public middle and high schools near major Adventist universities in Southern California and Michigan. A total of 601 adolescents (262 males and 339 females) aged 12–18 years (grades 7–12) participated in the study. Data were collected between February and May 2012 for southern California and between November 2012 and April 2013 for Michigan. Participants enrolled in the study with their parents who provided consent for their participation through a website created specifically for the study. A web-based survey developed for the study was used to collect information on dietary intake, lifestyle, and demographic information while anthropometric measurements were collected during school visits. All study protocols were approved by the Institutional Review Boards of Loma Linda University (IRB #5120014) and Andrews University (IRB Protocol #12-113).

### 2.2. Assessment of Milk and Dairy Products Intake

The web-based survey included a semi-quantitative food frequency questionnaire (FFQ) composed of 151 food items [38], 34 of which were milk- and/or dairy-containing foods. The FFQ included 10 dairy foods and 24 dairy-containing food items.

The USDA serving sizes were used for each food item. For example, 8 fluid ounces is one serving of milk, 6 oz. is one serving of yogurt or ice cream, and 1 ½ oz. is a serving of cheese. The research dietitians examined each of the dairy-containing food items, such as pizza, and approximated the amount of milk and/or dairy relative to the portion size specified in the FFQ for each of these foods. For example, the specified portion size for cheese pizza in the FFQ is medium size, and cheese from such a portion size is approximately 3 oz., which is equivalent to 2 servings.

Dairy food products were sorted into three categories: milk, cheese, and sweetened dairy. The milk group included 6 food items such as non-fat, low-fat, and whole milk, as well as the amount of milk added to mixed foods, such as mashed potatoes or cream soups. The cheese group consisted of 24 food items including different types of cheese and cheese found in convenience foods (e.g., egg and cheese breakfast sandwich, tacos, and pizzas) and mixed foods (e.g., pasta, soups, and snacks). The sweetened dairy group included 4 food items: yogurt, ice cream, milk-based smoothies, and blended coffee/frappuccino. Total dairy intake was computed as the sum of these three categories comprising a total of 34 food items. Intake of total dairy was categorized as low if consumption is less than 1 serving per day, medium if consumption is 1 to less than 3 servings per day, and high if consumption is 3 or more servings per day.

### 2.3. Assessment of Growth Parameters and Indicators of Obesity

Trained research assistants of TFADS collected the clinical data for anthropometric measurements during the designated school visits. A stadiometer (Seca Portable™, Chino, CA, USA) was used to measure height, and a bioelectric impedance analysis scale (TANITA™, model TBF-300A, Arlington Heights, IL, USA) was used to collect weight and body composition measures (fat mass and fat-free mass). Waist circumference was measured with a cloth measuring tape placed about midpoint between the lower margin of the last palpable rib and the top of the iliac crest.

The outcome variables were the growth parameters (age- and sex-specific WAZ, HAZ, and BMIZ) and indicators of obesity (BMIZ and WHtR), which have been determined to be appropriate for the adolescent population [39,40]. Due to a multi-ethnic study population, the WAZ, HAZ, and BMIZ were calculated based on World Health Organization (WHO) standards [40,41]. BMIZ was used as an indicator of general adiposity while WHtR was used as indicator of central adiposity [42]. Fat mass and fat-free mass were used as indicators of body composition.

### 2.4. Data Analysis

Sixty-five participants were excluded for various reasons, including improbable intake for either total calories or milk and dairy products, and/or because they did not have their anthropometrics data completed. Improbable caloric intake was defined as <500 kcal/day or >3500 kcal/day for girls and <800 kcal/day and >4500 kcal/day for boys, while improbable dairy intake was defined as more than 10.5 servings/day. Data from 536 participants (305 males and 231 females) were used in the final analytical dataset.

Data analysis was done using SPSS version 23 for Windows (IBM SPSS, Inc., Armonk, NY, USA). Descriptive analysis was performed for the demographic profile of the participants. Comparisons were done using either parametric (T-test and ANOVA) or non-parametric (Chi-square, Wilcoxon and/or Mann–Whitney U) tests depending on the type and distribution of the variables being analyzed. Categories of BMIZ were based on the WHO cut-off points: normal is z-score between ≥−2.0 and ≤+1.0 and overweight-obese BMIZ is z-score > +1.0 [40]. Categories for WHtR were based on the cutoff value of 0.5 where a value higher than 0.5 was used to define abdominal obesity [42,43].

Multiple linear regression analyses were used to determine associations between dairy intake and the anthropometric measures. Variables with non-normal distributions—total dairy, milk, cheese, and sweetened dairy—were log-transformed for these analyses. Due to inherent gender differences in fat mass, fat-free mass, and waist circumference, analyses for these variables were done gender specific. Models for the multiple linear regression adjusted for age, gender, ethnicity, energy intake, and mother’s educational level. Mother’s educational level was included in this first model because a few studies have shown it to be an independent risk factor for overweight/obesity [44,45,46]. Further adjustments for the same analyses were done for other variables that could confound associations between dairy intake and obesity including soda intake [17], physical activity [15,17], and milk substitute. Milk substitute intake was controlled for due to our observation that this beverage is commonly consumed in this adolescent population. An exploratory check showed milk substitute intake to be significantly, although weakly, correlated with milk (*r* = −0.31, *p* < 0.001), total dairy (*r* = −0.19, *p* < 0.001) and BMIZ (*r* = −0.10, *p* = 0.02).

## 3. Results

### 3.1. Demographic Profile of Participants

For reasons mentioned earlier, 536 subjects (231 (43.2%) males and 305 (56.8%) females) with mean (SD) age of 14.99 (1.70) years comprised the basis of all the analyses. Table 1 shows the characteristics of the study population in terms of demographic profile and other pertinent variables for all participants and according to gender and level of dairy consumption. The study population was a fairly diverse group with 37% Caucasian, 15% Hispanic, 13% Asian/Pacific Islander, 10% African/African-American, and 25% with other or mixed ethnicities. This population had highly educated parents with almost 50% of the mothers having at least a college degree and about 40% having graduate-level degrees. No significant differences were noted between males and females regarding age, ethnicity, parents’ ethnicity or educational level, mean energy intake distributions, or categories of BMIZ and WHtR.

Evaluating levels of dairy consumption, significantly more girls consumed low and medium levels of dairy compared to boys. There were also statistically significant distribution differences between participants’ mothers’ ethnicities and participants’ mean hours of physical activity per week. The mean caloric intake of the low dairy intake group was almost 1000 calories lower than that of the high dairy intake group (1800 vs. 2796 kcal) (*p* < 0.0001). Twenty-three percent of females and 21% of males were considered overweight or obese according to BMIZ score categories, and almost 13% of males and 20% of females were considered centrally obese according to WHtR categories. There were no significant distribution differences between the normal and overweight or obese participants except for the ethnicity of the father. More of the overweight or obese participants had fathers who were African/African-American or West Indian. Normal BMIZ participants were more likely to have Caucasian, Asian, or Pacific Islander fathers (not shown in Table 1).

### 3.2. Gender Comparison of Dietary Intake

The mean intake for total dairy was 3.0 (SD = 1.8) servings/day; the mean intake for milk was 0.7 (SD = 1.0) serving/day; the mean intake for cheese was 1.9 (SD = 1.29) servings/day; and the mean intake for sweetened dairy was 0.4 (SD = 0.5) serving/day. It should be noted that over 30% (*n* = 166) of the participants were non-consumers of milk, and another 27% (*n* = 147) consumed less than one cup per day. Dairy intake showed a high variability from non-consumers to very high consumers. For total dairy, 35% (*n* = 188) consumed less than one serving of dairy per day.

Boys drank significantly more milk (0.9 ± 1.2 serving/day) compared to girls (0.6 ± 0.6 serving/day) (*p* < 0.0001). The total dairy intake of boys, 3.4 ± 2.04 serving/day, was substantially higher than that of girls, 2.7 ± 1.6 serving/day (*p* < 0.0001). Cheese intake was also significantly higher in boys (2.1 ± 1.3 servings/day) compared to girls (1.7 ± 1.1 serving/day) at *p* = 0.001. Intake of sweetened dairy was similar for both genders.

In comparing mean intakes of dairy for normal-weight participants and their overweight or obese counterparts, the differences were not statistically significant. Normal-weight males consumed more milk than their overweight or obese counterparts (0.9 vs. 0.8 serving/day) but less total dairy (3.3 vs. 3.6 serving/day), cheese (2.0 vs. 2.3), and sweetened dairy (0.4 vs. 0.5) than their overweight or obese counterparts. The mean intakes of normal-weight and overweight/obese girls were very similar.

### 3.3. Anthropometric Parameters across Levels of Dairy Intake

Table 2 shows the means of the anthropometric growth parameters, obesity indicators, and body composition of all subjects according to level of dairy food intake. There were no significant trends in any of these anthropometric indicators as level of dairy intake increased. Although there seemed to be increasing trends in BMIZ and WAZ as total cheese intake increased and increasing trends in WAZ, HAZ, and fat-free mass as sweetened dairy intake increased, none were significant.

### 3.4. Associations between Dairy Intake and Measures of Growth, Adiposity and Body Composition

The results of multiple regression analyses for associations between intake of dairy foods and anthropometric indicators of growth (in z-scores) and central adiposity are presented in Table 3. The results showed a significant positive association between total dairy and WAZ (*p* = 0.045) both in the base and the fully-adjusted models. Total dairy was also significantly associated with HAZ in both models (*p* = 0.021). No significant associations were found between dairy intake and BMIZ or WHtR in any of the models. A large number of participants (*n* = 166) were non-consumers of milk (see Table 2) and were excluded during stratification according to milk type (non-fat, low-fat, and full-fat). The stratified analysis showed a significant association between low/reduced-fat milk intake and height-for-age in the fully adjusted model (see Table 3).

Table 4 shows the results of multiple regression analyses to determine associations between dairy intake and anthropometric parameters according to gender. In males, significantly positive associations were seen between total dairy intake and fat-free mass, fat mass, and WHtR, even in the fully-adjusted model. Milk intake was also positively significantly associated with WHtR in males only, but further adjustment for other confounders attenuated the association to non-significance (*p* = 0.055). Cheese intake was also positively significantly associated with fat-free mass (*p* = 0.008) in males even after controlling for all confounders. Type of milk was not associated with any of the parameters both in males and females. No significant associations were seen between intake of any type of dairy and the same anthropometric indicators of body composition in females.

## 4. Discussion

The purpose of this study was to determine the interrelationships among milk and dairy foods consumption and growth parameters and health indicators in an adolescent population known to follow a healthy lifestyle. Significant positive associations were seen between total dairy intake and HAZ and WAZ and remained so even after adjusting for all known confounders. In boys, significant positive associations were found between total dairy intake and WHtR, fat mass, and fat-free mass; cheese intake was also associated with fat-free mass for boys.

Total dairy intake in this population of adolescents was found to be significantly associated with both HAZ and WAZ, which were used in this study as indicators of growth. The relationship between total dairy and growth indicators could be explained in two ways: (1) dairy could promote growth since it is a good source of protein; or (2) physical growth demands increased intake energy and foods, in particular foods that can support linear or bone growth, such as dairy. [7] As a rich source of calcium and protein, dairy was found to be positively associated with the bone mineral content of girls in their middle childhood [47] and of adolescent girls [48]. Higher intake of dairy foods was also found to be associated with bone growth and height of black and white adolescents [49]. The connection between dairy products and physical stature has been of significant interest to researchers [50,51,52,53,54,55]. Our study adds to this important area. As discussed earlier, our study found that higher dairy intake contributed to significantly higher HAZ in both genders. Reviewing a meta-analysis on the relationship between dairy products and physical stature supports moderate quality evidence that dairy products can actually stimulate linear growth [54].

Intake of low-fat milk showed significant association with HAZ but this was only after controlling for all confounders, and thus, could be a spurious relationship considering repeated analyses on the same data and a sample size that had become even smaller after stratification and exclusion of non-consumers of dairy milk.

Dairy intake seems to play a role in the tendency for central adiposity and body composition of boys but not girls. Total dairy intake in boys is positively associated with WHtR, fat mass, and fat-free mass. Milk and cheese intake seem to be responsible for these associations. Similar to our findings, Snijder et al. in the Hoorn study, found no significant association between dairy consumption and BMI. However, cheese was positively associated with BMI [21]. Wiley evaluating the relationship between milk and dairy with BMI among children 2–4 and 5–10 years of age found that those in the highest quartile of milk intake had higher BMI than those consuming less milk [50]. Berkey following a cohort of 12,829 of children 9–14 years found that drinking large amounts of milk may provide increased energy and showed increased overall weight in the adolescent population [23].

Furthermore, in the regression analyses used to determine associations between dairy intake and body composition, the point estimate for cheese—β value—is higher compared to the point estimates for milk and sweetened dairy, the other components of total dairy intake (see Table 4). While the relationship between total dairy intake and body composition in boys may not be attributed to only one type of dairy food, it seems that cheese is the driving force. The relationship between total dairy intake and body composition in boys could be explained in two ways: (1) there are biological differences in response to dairy intake (e.g., dairy intake is only related to body composition in boys); or (2) there is more variability in intake of dairy in boys than in girls. Other explanation for the relationship found between cheese intake and body composition in this adolescent population could be investigated further, which includes fat content of cheese or foods associated with cheese intake.

Dairy provides a good source of protein and because males have more muscle mass than females, growing adolescent males may be increasing muscle mass by eating dairy foods [19,20]. Some dairy is also high in fats (e.g., whole fat varieties) and thus may affect an adolescent male’s body fat composition and distribution. However, this might not be the case for adolescent females who consumed less total dairy and cheese (but more sweetened dairy) than males. The greater variability of dairy intake in boys could be the reason why we found significant findings in this gender group. Another possible explanation for the differences between genders could be the visceral and subcutaneous fat differences that exist between genders [55]. These factors, along with hormonal changes that occur during growth and puberty and the effects of calcium on cortisol production and visceral adipocytes stimulation, is not fully examined in children and adolescents [14]. This lack of information makes this issue more complicated to evaluate [56,57,58].

Our main findings that dairy consumption is not associated with general body obesity are consistent with multiple cross sectional studies. Forshee found no association between BMI with milk consumption [59]. Fiorito evaluating the relationship among girls’ weight status and dairy found no association between dairy intake and body weight or percent body fat [47]. Furthermore, Phillips et al. found no significant correlations between dairy intake and BMIZ or percentage of body fat [60]. Lin et al. did not find associations between dairy products and lower adolescent BMI or WHtR even after adjusting for confounders [26]. When evaluating randomized controlled trials that evaluated the effects of dairy, calcium, and milk supplementation on overweight and obesity in adolescent girls, we also see similar results [56,57,58]. Lastly, our finding is consistent with most of the longitudinal studies reviewed by Louie et al. [29]. Out of the 10 studies reviewed, six showed no significant association, one with an increased risk of obesity, and three with a protective association between dairy consumption and risk of overweight or obesity [29].

Contrary to our findings, Abreu et al. found an inverse association between dairy products and abdominal obesity in Azorean adolescent boys [14]. Using the same cross-sectional data, Abreu et al. also found an inverse association between milk intake and BMI and body fat percentage in girls [13]. Similarly, Moore et al. found a significant positive association between lower dairy intake and BMI as well as increased subcutaneous fat [19].

Overall, our data do not support the hypothesis that consuming higher amounts of dairy is associated with a decreased likelihood of becoming overweight, at least for adolescents. Further, dairy intake seems to play a role in the tendency for central adiposity and body composition of boys, but not girls.

### Limitations of the Study

Our study is not without limitations. The original research design of this study was cross-sectional which means no conclusions related to causation can be drawn. Furthermore, self-reported FFQs completed by participants may be subject to recall error. In general, FFQs can have substantial errors including the difference between estimated portion sizes and the total amount of food actually consumed. And, while FFQs are useful for ascertaining the types and amounts of food consumed and estimating habitual and relative caloric intake, researcher-created FFQs cannot capture all of the foods consumed and adequately reflect what the participants eat that might affect the outcome.

Although data were collected for two groups through two sites and our analyses controlled for each site, our analyses do not take into account possible dietary differences between the two groups. Also, we found that our two-site population has lower rates of overweight and obesity compared to the U.S. adolescent population, which leaves us with a small number of overweight/obese subjects (22%) ,which could affect further the power to see significant findings.

Our stratified analysis for non-fat, low-fat, and full-fat versions of milk did not yield significant results. Considering that about one-third of our study population did not consume dairy milk, this could have limited our power to see if there was indeed an association between specific milk types based on fat content and certain anthropometric parameters in this population. However, it was not the intent of our study to investigate associations between dairy foods with varying fat contents and indicators of obesity. Thus, we categorized dairy foods known to be consumed by adolescent populations regardless of fat content.

While physical activity and soda intake were controlled for in our study, our study does not exclude the possibility that total dairy intake may be a proxy indicator of a generally healthier dietary pattern in this population. The validity of the adjustment for total energy intake in regression models needs to be carefully considered when interpreting study results and conducting cross-study comparisons. Given the high degree of correlation between total energy intake and overweight, adjustment for total energy intake appears appropriate.

Finally, it has been suggested that dairy products may reduce body fat via the effects of calcium [61]. It is possible that dietary supplements supply the dietary calcium, though supplement intake was not taken into consideration in our analysis nor did we evaluate calcium intake of this population

## 5. Conclusions

We found dairy intake to be significantly associated with growth in this adolescent population. We also found total dairy intake to be positively associated with WHtR, fat mass, and fat-free mass but only in boys. The role of dairy intake in the tendency for central adiposity and body composition seems to be gender specific. Milk and cheese intake seem to be responsible for these associations. Our study populations include approximately 20% vegetarians who consume varying amounts of milk and dairy foods together with or without milk substitutes, e.g., soy milk. Replication of our study in other adolescent populations would be useful to better understand observed associations. Assessing longitudinal changes in obesity-related indicators with dietary intake of milk and dairy foods would also be useful.

## Figures and Tables

**Table 1:** Demographic profile of subjects and distribution according to level of dairy consumption (*n* = 536).

**Demographic Variable**	**All Subjects** ***n* (%)**	**Males** ***n* (%)**	**Females** ***n* (%)**	**M vs. F** *p* *	**Total Dairy Intake Level**			p *
					**Low (<1 svg/Day)**	**Med (1 to <3 svg/Day)**	**High (3+ svg/Day)**	
**Gender**				0.589				<0.0001
Female			305 (56.8)		123 (22.9)	108 (20.1)	74 (13.8)	
Male		231 (43.20)			65 (12.1)	74 (13.8)	92 (17.2)	
**Age, years**				0.865				0.636
12	38 (7.1)	14 (6.1)	24 (7.9)		10 (5.4)	14 (7.7)	14 (8.4)	
13	91 (17.0)	43 (18.6)	48 (15.7)		36 (19.1)	27 (14.8)	28 (16.9)	
14	90 (16.8)	36 (15.6)	54 (17.7)		32 (17)	28 (15.4)	30 (18.1)	
15	100 (18.7)	45 (19.5)	55 (18.0)		33 (17.6)	39 (21.4)	28 (16.9)	
16	98 (18.3)	42 (18.2)	56 (18.0)		30 (16)	41 (22.5)	27 (16.3)	
17	77 (14.4)	31 (13.4)	46 (15.1)		30 (16)	22 (12.1)	25 (15.1)	
18	42 (7.9)	20 (8.6)	22 (7.3)		17 (9.1)	11 (6.0)	14 (8.4)	
**Ethnicity (child)**				0.551				0.102
African/Afr-Am	50 (9.6)	23 (4.4)	27 (5.2)		25 (31.9)	14 (7.9)	11 (6.9)	
Caucasian	192 (37.0)	89 (17.1)	103 (19.8)		58 (11.2)	64 (36.2)	70 (43.8)	
Hispanic	76 (14.6)	34 (6.6)	42 (8.1)		24 (13.2)	28 (15.8)	24 (15.0)	
Asian	67 (12.9)	29 (5.6)	38 (7.3)		29 (15.9)	19 (10.7)	16 (10.0)	
Others	37 (7.1)	13 (2.5)	24 (4.6)		13 (7.1)	10 (5.6)	14 (8.8)	
Mixed ethnicities	97 (18.7)	35 (6.7)	62 (11.9)		33 (18.1)	42 (23.7)	25 (16.6)	
**Mother’s ethnicity**				0.429				0.026
African/Afr-Am	55 (10.6)	24 (10.8)	31 (10.5)		27 (14.8)	15 (8.5)	13 (8.1)	
Caucasian	221 (42.6)	100 (45)	121 (41)		62 (34.1)	81 (45.8)	78 (48.8)	
Hispanic	102 (19.7)	41 (18.4)	61 (21)		32 (17.6)	39 (22)	31 (19.4)	
Asian	88 (17)	41 (18.4)	47 (15.9)		42 (23.1)	26 (14.7)	20 (12.5)	
Others	53 (10.2)	17 (7.6)	36 (12.2)		19 (10.4)	16 (9)	18 (11.3)	
**Father’s ethnicity**				0.826				0.100
African/Afr-Am	64 (12.5)	27 (12.1)	38 (12.8)		32 (17.6)	21 (11.9)	12 (7.5)	
Caucasian	227 (33.7)	103 (46.2)	124 (41.9)		76 (41.8)	75 (42.4)	76 (47.5)	
Hispanic	96 (18.5)	43 (18.5)	56 (19.2)		26 (14.3)	38 (21.5)	32 (20)	
Asian	74 (14.3)	31 (13.9)	43 (14.5)		31 (17)	24 (13.6)	19 (11.9)	
Others	57 (11.0)	21 (9.4)	36 (12.2)		17 (9.3)	19 (10.7)	21 (13.1)	
**Mother’s Educ Level**				0.928				0.189
HS or less	74 (15.3)	31 (13.9)	43 (14.5)		18 (9.9)	26 (14.7)	30 (18.8)	
Some College/Grad	245 (47.2)	104 (46.6)	141 (47.6)		87 (47.8)	83 (46.9)	75 (46.9)	
Graduate level	200 (38.5)	88 (39.5)	112 (37.8)		77 (42.3)	68 (38.4)	55 (34.4)	
**Father’s Educ Level**				0.123				0.091
HS or less	93 (17.9)	46 (20.6)	47 (15.9)		26 (14.3)	30 (16.9)	37 (23.1)	
Some College/Grad	194 (37.4)	73 (32.7)	121 (40.9)		70 (38.5)	60 (33.9)	64 (40)	
Graduate level	232 (44.7)	104 (46.6)	12 (43.2)		86 (47.3)	87 949.2)	59 (36.9)	
**Site**				0.252				0.946
California	299 (55.8)	135 (58.4)	164 (54)		105 (55.9)	103 (56.6)	91 (54.8)	
Michigan	237 (44.2)	96 (41.6)	141 (46)		83 (44.1)	79 (43.4)	75 (45.2)	
**BMI z-score ^a^**				0.589				0.820
Normal	414 (77.7)	182 (44.0)	232 (56.0)		148 (78.7)	137 (76.1)	129 (78.2)	
Overwt/Obese	119 (22.3)	49 (41.2)	70 (58.8)		40 (21.3)	43 (29.9)	36 (21.8)	
**Waist-to Height Ratio (WHtR) ^b^**				0.406				0.411
Normal	431 (83.4)	199 (87.3)	232 (80.3)		148 (80.9)	145 (28)	138 (86.3)	
Overweight/Obese	86 (16.6)	29 (12.7)	57 (19.7)		35 (19.1)	29 (16.7)	22 (13.8)	
**Other Variables**	**All Subjects**	**Males**	**Females**	**M vs. F** *p* **	**Total Dairy Intake Level**			p ***
					**Low (<1 svg/Day)**	**Med (1 to <3 svg/Day)**	**High (3+ svg/Day)**	
	**Mean (SD) or Median (Q1, Q3) ^c^**				**Mean (SD) or Median (Q1, Q3) ^c^**			
**Age**, years	15.0 (1.7)	15.0 (1.7)	15.0 (1.8)	0.817	15.0 (1.8)	15.0 (1.6)	14.9 (1.8)	
**Energy**, kcal	2198 (816)	2335 (837)	2093 (784)	0.481	1800 (698)	2061 (623)	2790 (788)	<0.001
**Soda**, svg/day ^c^	0.07 (0.00, 0.14)	0.07 (0.07, 0.21)	0.07 (0.00, 0.14)	<0.001	0.07 (0.00, 0.13)	0.07 (0.07, 0.14)	0.13 (0.07, 0.32)	<0.001
**Milk substitutes**, svg/day ^c^	0.07 (0.00, 0.79)	0.13 (0.00, 0.79)	0.07 (0.00, 0.79)	0.840	0.43 (0.00, 1.00)	0.07 (0.00, 0.56)	0.00 (0.00, 0.32)	<0.001
**Phys Act**, min/day ^c^	26.0 (11.3, 51.4)	26.0 (12.8, 51.4)	26.0 (9.4, 42.2)	<0.001	26.0 (9.7, 42.2)	26.0 (12.8, 51.4)	26.0 (9.7, 51.4)	0.004
**Sleep**, h/night	7.75 (1.24)	8.00 (1.25)	7.56 (1.20)	<0.001	7.63 (1.25)	7.74 (1.19)	7.89 (1.26)	0.160

* Comparisons by Chi-square ** Comparison by independent T-test or Mann–Whitney U; *** Comparison by ANOVA or Kruskal–Wallis; ^a^ Z-score cut off values are: Normal weight: z ≥ −2.0 to ≤ +1.0, Overweight: z > +1.0 to ≤ +2.0; Obese: z > +2.0; ^b^ WHtR higher than 0.5 is considered abdominal obesity; ^c^ Variable is non-normally distributed so median values are reported; Q1 is quartile 1 or 25th percentile and Q3 is third quartile or 75th percentile.

**Table 2:** Mean (95% CI) of growth and health indicators according to levels of dairy foods intake, all subjects.

Mean Intake (95% CI)	BMIZ *	WAZ *	HAZ *	WHtR *	Fat-Free Mass, kg	Fat Mass, kg
**Total Dairy**						
Low (< 1 svg/day *), *n* = 188	0.24 (0.08, 0.39)	0.21 (0.06, 0.36)	−0.06 (−0.21, 0.08)	0.46 (0.45, 0.47)	46.42 (45.41, 47.43)	12.609 (11.36, 13.86)
Medium (1 to <3 svg/day), *n* = 181	0.29 (0.14, 0.43)	0.39 (0.24, 0.52)	0.17 (0.03, 0.32)	0.46 (0.45, 0.46)	47.37 (46.42, 48.32)	13.207 (12.03, 14.38)
High (3+ svg/day), *n* = 166	0.26 (0.09, 0.44)	0.37 (0.20, 0.54)	0.15 (−0.13, 0.32)	0.46 (0.45, 0.47)	46.69 (45.57, 47.82)	13.69 (12.29,15.09)
*p*-trend	0.79	0.17	0.50	0.92	0.63	0.29
**Cheese**						
Low(< 1 svg/day), *n* = 130	0.24 (0.09, 0.40)	0.25 (0.11, 0.41)	−0.027 (−0.18, 0.13)	0.46 (0.45, 0.47)	46.59 (45.56, 47.62)	13.28 (12.00, 14.57)
Medium (1 to <3 svg/day), *n* = 224	0.25 (0.10, 0.40)	0.33 (0.19, 0.48)	0.169 (0.03, 0.31)	0.45 (0.45, 0.46)	46.97 (46.00, 47.94)	12.64 (11.44, 13.84)
High (3+ svg/day), *n* = 181	0.29 (0.14, 0.45)	0.36 (0.21, 0.52)	0.11 (−0.04, 0.26)	0.46 (0.45, 0.47)	46.92 (45.90, 47.96)	13.50 (12.22, 14.78)
*p*-trend	0.71	0.37	0.23	0.97	0.66	0.88
**Sweetened Dairy**						
Low(< 1 svg/day), *n* = 130	0.26 (0.11, 0.40)	0.29 (0.15, 0.44)	0.03 (−0.11, 0.18)	0.46 (0.45, 0.47)	46.66 (45.70, 47.63)	13.31 (12.12, 14.51)
Medium (1 to <3 svg/day), *n* = 224	0.27 (0.14, 0.40)	0.32 (0.19, 0.45)	0.06 (−0.06, 0.20)	0.46 (0.45, 0.49)	46.78 (45.91, 47.63)	13.20 (12.12, 14.27)
High (3+ svg/day), *n* = 181	0.25 (0.08, 0.43)	0.35 (0.18, 0.53)	0.18 (0.01, 0.36)	0.45 (0.45, 0.47)	47.17 (46.00, 48.34)	12.81 (11.37, 14.27)
*p*-trend	0.98	0.62	0.21	0.75	0.53	0.62
**Milk**						
Non-consumers, *n* = 166	0.16 (0.00, 0.31)	0.23 (0.08, 0.38	0.08 (−0.07, 0.23)	0.46 (0.45, 0.47)	46.65 (45.65, 47.67)	12.73 (11.48, 13.99)
Low (< 1 cp/day), *n* = 147	0.28 (0.12, 0.44)	0.30 (0.14, 0.46)	0.03 (−0.13, 0.19)	0.45 (0.44, 0.46)	47.23 (46.17, 48.29)	13.03 (11.72, 14.35)
Medium (1 to <2 cp/day), *n* = 80	0.37 (0.15, 0.59)	0.42 (0.21, 0.64)	0.09 (−0.13, 0.30)	0.46 (0.45, 0.48)	46.52 (45.08, 47.98)	13.68 (11.88, 15.48)
High (2+ cps/day), *n* = 142	0.31 (0.14, 0.48)	0.39 (0.22, 0.55)	0.15 (−0.02, 0.31)	0.46 (0.45, 0.47)	46.80 (45.67, 47.93)	13.45 (12.06, 14.86)
*p*-trend	0.17	0.14	0.51	0.48	0.98	0.39

* svg/day = serving per day; cp/day = cup per day (1 cup = 8 fl. oz.); BMIZ= body mass index z-score; WAZ = weight-for-age z-score; HAZ = height-for-age z-score; WHtR = waist-to-height ratio.

**Table 3:** Associations * between types of dairy foods and anthropometric indicators of growth (in z-scores) and central adiposity.

Dairy Food	BMIZ *			WAZ *			HAZ *			WHtR *		
	β	95% CI	*p*	β	95% CI	*p*	β	95% CI	*p*	β	95% CI	*p*
**Basic Model ^a^**												
Total Dairy, serving/day	0.16	−0.08, 0.39	0.186	0.30	0.07, 0.53	0.010	0.27	0.04, 0.50	0.021	1.40	−1.02, 3.83	0.255
Milk, serving/day	0.13	−0.07, 0.34	0.199	0.17	−0.29, 0.37	0.094	0.12	−0.08, 0.32	0.226	0.78	−1.33, 2.90	0.467
Full fat	−0.04	−0.18, 0.09	0.514	0.01	−0.12, 0.15	0.840	0.11	−0.02, 0.24	0.085	−0.00	−0.01, 0.00	0.310
Low-fat	0.02	−0.09, 0.13	0.730	0.07	−0.04, 0.17	0.229	0.09	−0.01, 0.20	0.082	0.00	−0.01, 0.01	0.655
Non-fat	0.14	−0.03, 0.30	0.109	0.08	−0.08, 0.24	0.31	−0.11	−0.25, 0.04	0.142	0.01	−0.00, 0.02	0.137
Cheese, serving/day	0.11	−0.15, 0.37	0.392	0.23	−0.02, 0.49	0.071	0.20	−0.05, 0.45	0.122	0.88	−1.79, 3.54	0.519
Sweetened dairy, serving/day	0.11	−0.25, 0.49	0.530	0.23	−0.13, 0.58	0.205	0.28	−0.06, 0.63	0.114	1.21	−2.51, 4.93	0.522
**Fully Adjusted Model ^a^**												
Total Dairy, serving/day	0.08	−0.17, 0.33	0.519	0.25	0.01, 0.49	0.045	0.28	0.04, 0.52	0.021	0.00	−0.01, 0.02	0.808
Milk, serving/day	0.08	−0.14, 0.29	0.472	0.13	−0.08, 0.33	0.236	0.13	−0.08, 0.33	0.240	0.00	−0.01, 0.01	0.843
Full-fat	−0.05	−0.19, 0.08	0.445	0.01	−0.13, 0.15	0.878	0.12	−0.01, 0.25	0.066	−0.01	−0.01, 0.00	0.286
Low-fat	0.02	−0.09, 0.13	0.678	0.08	−0.03, 0.18	0.158	0.12	0.01, 0.23	0.036	0.00	−0.01, 0.01	0.691
Non-fat	0.14	−0.03, 0.31	0.102	0.07	−0.09, 0.23	0.309	−0.12	−0.27, 0.03	0.127	0.01	−0.00, 0.02	0.098
Cheese, serving/day	0.09	−0.17, 0.35	0.505	0.21	−0.05, 0.46	0.107	0.20	−0.06, 0.45	0.129	0.00	−0.02, 0.02	0.891
Sweetened dairy, serving/day	0.12	−0.25, 0.48	0.530	0.28	−0.08, 0.63	0.122	0.28	−0.08, 0.63	0.127	0.00	−0.02, 0.02	0.873

* Multiple linear regression analyses used to determine relationships; BMIZ = body mass index z-score; WAZ = weight-for-age z-score; HAZ = height-for-age z-score; WHtR = waist-to-height ratio; **^a^** Basic Model adjusted for age, site, ethnicity, education of mother, and energy intake; Fully-adjusted model includes the basic model plus additional adjustment for soda intake, physical activity, and milk substitutes intake (for total dairy and milk only).

**Table 4:** Association * between types of dairy foods intake (servings/day) and body composition and indicator of central adiposity, by gender.

Dairy Food	Male									Female								
	Fat-Free Mass			Fat Mass			WHtR ^b^			Fat-Free Mass			Fat Mass			WHtR ^b^		
	β	95% CI	*p*	β	95% CI	*p*	β	95% CI	*p*	β	95% CI	*p*	β	95% CI	*p*	β	95% CI	*p*
**Basic Model ^a^**																		
Total Dairy	4.68	1.76, 7.60	0.002	4.03	0.83, 7.23	0.014	0.03	0.01, 0.05	0.019	0.00	−1.45, 1.45	0.998	0.14	−2.31, 2.59	0.913	−0.01	−0.03, 0.01	0.425
Milk	1.22	−1.01, 3.44	0.283	2.21	−0.19, 4.62	0.071	0.02	0.00, 0.04	0.030	−0.58	−1.99, 0.84	0.424	−0.66	−3.06, 1.73	0.587	−0.01	−0.03, 0.01	0.169
Full-fat	−0.01	−1.64, 1.63	0.993	0.37	−1.84, 2.58	0.739	0.00	−0.01, 0.01	0.829	0.09	−0.85, 1.04	0.840	−0.43	−1.98, 1.13	0.588	−0.01	−0.02, 0.00	0.108
Low-fat	0.25	−0.94, 1.45	0.674	0.23	−1.24, 1.69	0.759	0.00	−0.01, 0.01	0.932	0.35	−0.47, 1.17	0.401	0.45	0.87, 1.76	0.502	−0.00	−0.01, 0.02	0.451
Non-fat	0.46	−1.29, 2.21	0.599	1.71	−1.60, 5.01	0.298	0.01	−0.01, 0.03	0.381	−0.48	−1.73, 0.77	0.437	0.22	−1.83, 2.27	0.831	0.01	−0.00, 0.03	0.354
Cheese	4.35	1.13, 7.56	0.008	2.57	−0.96, 6.10	0.153	0.01	−0.01, 0.04	0.406	0.24	−1.36, 1.83	0.769	1.26	−1.42, 3.95	0.355	0.00	−0.02, 0.02	0.935
Sweetened dairy	3.58	−0.41, 7.56	0.078	1.05	−3.31, 5.41	0.636	0.01	−0.02, 0.04	0.463	0.55	−2.01, 3.11	0.674	−1.64	-5.97, 2.69	0.456	−0.01	-0.04, 0.02	0.633
**Fully Adjusted Model ^a^**																		
Total Dairy	4.83	1.79, 7.87	0.002	3.89	0.58, 7.21	0.021	0.02	0.00, 0.04	0.039	−0.28	−1.85, 1.30	0.731	−0.62	−3.27, 2.02	0.642	−0.01	−0.03, 0.00	0.222
Milk	1.10	−1.21, 3.41	0.350	2.07	−0.41, 4.55	0.102	0.01	0.00, 0.03	0.055	−0.76	−2.24, 0.71	0.311	−1.18	−3.66, 1.30	0.351	−0.01	−0.04, 0.00	0.095
Full-fat	−0.06	−1.76, 1.63	0.941	0.18	−2.12, 2.48	0.876	0.00	−0.01, 0.01	0.988	−0.06	−0.96, 1.07	0.908	−0.94	−2.60, 0.72	0.260	−0.01	−0.03, 0.00	0.07
Low-fat	0.27	−0.96, 1.50	0.667	0.24	−1.27, 1.75	0.754	0.00	−0.01, 0.01	0.883	0.49	−0.34, 1.32	0.241	0.58	−0.71, 1.89	0.372	0.01	−0.01, 0.02	0.377
Non-fat	0.09	−1.79, 1.97	0.924	2.62	−1.03, 6.26	0.152	0.01	−0.01, 0.03	0.208	−0.59	−1.94, 0.77	0.383	−0.15	−2.26, 1.96	0.887	−0.01	−0.01, 0.03	0.360
Cheese	4.34	1.12, 7.56	0.008	2.48	−1.03, 5.99	0.164	0.00	−0.02, 0.03	0.472	−0.06	−1.67, 1.54	0.941	0.72	−1.97, 3.42	0.598	−0.00	−0.02, 0.02	0.831
Sweetened dairy	3.75	−0.24, 7.74	0.066	0.94	−3.39, 5.28	0.667	0.01	−0.02, 0.04	0.467	0.28	−2.28, 2.84	0.827	−2.12	−6.42, 2.17	0.331	−0.01	−0.04, 0.02	0.534

* Determined using multiple regression analysis; **^a^** Basic Model adjusted for age, site, ethnicity, education of mother, and energy intake; Fully-adjusted model includes the basic model plus total soda intake (servings/day), physical activity, and milk substitutes; ^b^ WHtR is waist-to-height ratio.
